# Investigation on the Recovery of Rare Earth Fluorides from Spent Rare Earth Molten Electrolytic Slag by Vacuum Distillation

**DOI:** 10.3390/ma18071538

**Published:** 2025-03-28

**Authors:** Ziyan Yang, Faxin Xiao, Shuchen Sun, Ganfeng Tu, Zhentao Zhou, Jingyi Chen, Xin Hong, Wei He, Chengfu Sui, Kuopei Yu

**Affiliations:** 1 School of Metallurgy, Northeastern University, Shenyang 110819, China; 2110602@stu.neu.edu.cn (Z.Y.); sunsc@smm.neu.edu.cn (S.S.); 2310687@stu.neu.edu.cn (Z.Z.); 2210589@stu.neu.edu.cn (J.C.); 2271662@stu.neu.edu.cn (X.H.); 20213335@stu.neu.edu.cn (W.H.); 2 Key Laboratory for Recycling of Nonferrous Metal Resources (Shenyang), Shenyang 110819, China; 3 Qingdao Qingli Environmental Protection Equipment Co., Ltd., Qingdao 266300, China; suichengfu@qingli.cn (C.S.); yukuopei@qingli.cn (K.Y.)

**Keywords:** rare earth molten electrolytic slag, rare earth fluorides, vacuum distillation, recycling

## Abstract

Spent rare earth molten salt electrolytic slag (REMES) needs to be recovered not only because of its economic value of rare earth elements (REEs), lithium, and fluorine, but also for the environmental benefits. Vacuum distillation has many advantages, such as a short process and less wastewater. Aiming to find an environmentally friendly method to recover REEs, this research studied the challenges in recovering REMES by vacuum distillation and the solutions to handle these obstacles. Distillation experiments for the raw material were initially implemented and XRD, XPS, DSC, and SEM methods were used to investigate the phase changes of REMES, thus discovering that oxide impurities could transform REF_3_ into REOF, which significantly affected the REEs recovery. Only 42.04% of the REEs could be evaporated at 1573 K and 0.1 Pa for 4 h with 99.99% of LiF. To tackle this issue, a fluorination pretreatment was proposed. NH_4_HF_2_ was utilized to transform oxide impurities, RE_2_O_3_, and REOF to fluorides with almost no waste gas released, significantly improving the recovery efficiency of the REEs, which was 86.23%. Therefore, this paper proposes this fluorination–vacuum distillation method, which has a short process to recover REF_3_ from REMES efficiently with almost no wastewater or gas released.

## 1. Introduction

Rare earth elements have some excellent chemical and physical properties, such as light, magnetism, and catalytic and electronic performance, achieving wide applications in many fields, such as electronic components, national defense, and aerospace [1,2,3,4,5,6,7]. With the market demand increasing, the annual production of rare earth oxides has increased from 210,000 to 350,000 tons over the past 5 years [8,9], leading to an increase in rare earth secondary resources. Many conspicuous secondary resources belong to post-consumer recycling [10], like waste permanent magnets and waste polishing powders [11,12,13,14]. In the pre-consumer recycling field, some resources, like red mud [15,16], have the potential to be recovered. Rare earth molten salt electrolysis slag is a typical example in the pre-consumer recycling field.

The main rare earth production method is rare earth fluoride molten salt electrolysis [17]. In this procedure, approximately 8% of REEs remain in the REMES and about 2200 tons of REMES will be produced each year [18,19]. Therefore, with the expansion of rare earth production, more REMES will be generated. Meanwhile, there is an abundance of valuable elements in REMES, including 10–60% of REEs, 10–25% fluorine, and 2–5 % lithium [19]. Therefore, it is essential to recycle it.

Due to the presence of fluorine, it is difficult to recover REMES directly by acid leaching. Therefore, the main challenge in recovery methodologies was to manage the fluorine and defluorination via phase reconstruction methods was studied initially [20]. Mubula et al. [21] categorized the recovery methodologies of REMES into three types, including alkali mineral phase reconstruction methods, salt mineral phase reconstruction methods, and acid mineral phase reconstruction methods [22,23,24]. Yang et al. [25] utilized sodium hydroxide to reconstruct the phases of rare earth compounds and lithium fluoride, transforming them into hydroxides. After the washing and leaching treatments, the optimal leaching efficiencies of the rare earths, fluorine, and lithium were 99.05%, 98.23%, and 99.22%, respectively. Yang [26] utilized borax to reconstruct REMES, generating rare earth oxides and sodium fluoride. After washing and leaching, the leaching efficiency of the REEs exceeded 97%. Tian et al. [27] proposed a method to reconstruct REMES using concentrated sulfuric acid. During the phase reconstruction process, the rare earth compounds and lithium fluoride were converted into sulfates, which would dissolve in water before the next step. Meanwhile, the released HF was collected and utilized to produce hydrofluoric acid. Then, the sulfate solution and hydrofluoric acid were mixed, precipitating rare earth fluorides and lithium fluoride from the solution. In this method, more than 90% of the REEs, Li, and F could be recovered. Additionally, some physical technologies were also used in the recovery of REMES, like microwaves and external electric fields [28,29]. Mubula et al. [29] used NaOH to transform rare earth fluoride into acid-soluble rare earth compounds, assisted with a microwave. Under optimal conditions, the conversion rate of rare earth fluorides reached 99.17%.

With the development of these methodologies, many issues have been handled, such as the recovery of lithium and the increase in resource utilization rates, and comprehensive resource recovery has been achieved, including the recovery of Li, F, and REEs. However, because of the defluorination process, the hydrometallurgical process was still the main purification method [30]. This would result in wastewater problems and a long production process, increasing the risk of environmental pollution and production costs. Lai [31] attempted to apply the vacuum distillation method to the recovery of REMES, while vacuum distillation was only utilized to extract LiF, and the extraction of REEs still involved a series of hydrometallurgical methods. This method successfully recovered fluorine, lithium, and REEs with reduced wastewater, while the hydrometallurgical method was still used in the recovery of REEs, and wastewater could still be generated in the recovery process.

Considering the volatility of rare earth fluorides and the cleanness of vacuum distillation, if vacuum distillation could also be used to recover rare earth fluorides, the generation of wastewater and gas might be reduced. The main traditional generation processes of REF_3_ and LiF are producing fluoride precipitation in solution or fluorinating raw materials by gaseous HF [32]. Although the energy consumption of vacuum distillation might be higher than that of these hydrometallurgical methods, the absence of wastewater treatment and the development of clean energy can achieve environmentally friendly resource recovery. However, the lack of saturated vapor pressure data for REF_3_ and the unknown phase transitions of REMES under high-temperature and -vacuum conditions limit the utilization of vacuum distillation. Therefore, this paper explored the challenges of introducing vacuum distillation to recover rare earth fluorides from REMES and proposed potential solutions to address these challenges.

## 2. Materials and Methods

### 2.1. Materials

This REMES was obtained from a rare earth factory in Jiangxi Province of China (JL MAG rare-earth Co., Ltd., Ganzhou, China). The valuable resources of REMES were mainly composed of rare earth fluorides, rare earth oxyfluorides, and lithium fluoride. NH_4_HF_2_ (98.5%, AR, Shanghai Macklin Biochemical Technology Co., Ltd., Shanghai, China) was selected as the fluorination reagent. The equipment in this study is a self-made vertical furnace. The furnace is composed of a cylindrical alundum tube, a heating system, and a vacuum system. The alundum tube is 1 m high with an inner diameter of 90 cm. The heating system consists of 6 silicon carbide rods, which are set around the alundum tube. The function of the vacuum system is provided by a vacuum pump called “First FX 32” (Shanghai First Vacuum Pump Co., Ltd., Shanghai, China).

### 2.2. Methods

Initially, the phase changes of REMES under high-temperature and -vacuum conditions were investigated to find the possible obstacles to recovering REF_3_ by vacuum distillation. The REMES was compacted as a block with no pretreatment and set at the center of the vertical vacuum distillation furnace. Then, many 10 g REMES blocks were distilled at different temperatures for different times. Based on the distillation results, the possible phase changes and potential obstacles were identified. Then, to resolve the barriers, the fluorination method was proposed, and NH_4_HF_2_ was selected as the fluorination reagent. It reacted with REMES in an airtight furnace. After the reaction, the fluorinated REMES was distilled to prove the availability of the fluorination and vacuum distillation. The recovery efficiencies of the REEs and Li were calculated by Equation (1), using the recovery efficiency of the REEs as an example.

(1) $$\eta_{N}=[1-(w_{NC}\times m_{C})/(w_{NR}\times m_{R})]\times 100\%$$

where $\eta_{N}$ represents the recovery efficiency of the REEs, %; $w_{NC}$ is the content of the REEs (calculated as oxides) in the distillation residue, %; $m_{C}$ is the mass of the distillation residue, g; $w_{NR}$ represents the content of the REEs (calculated as oxides) in the raw material, %; $m_{R}$ is the mass of the raw material, g.

Differential scanning calorimetry and thermo-gravimetric analysis were carried out by SDT (TA Q600, TA, New Castle, DE, USA) at a temperature range of 294–1466 K and a constant heating rate of 10 K/min in a N_2_ atmosphere. The phase analysis of the sample was conducted by an X-ray diffractometer (D8 Advance, Bruker, Bremen, Germany; ultima IV, Rigaku, Akishima, Japan), which used Cu-Ka radiation with a scanning rate of 10 °/min, a step size of 0.02°, and a recorded range from 10° to 90°. An inductively coupled plasma optical emission spectrometer (PE optima 8300DV, PerkinElmer, Waltham, MA, USA) was employed to analyze the concentration of Li. The XPS analysis was conducted by Thermo SCIENTIFIC ESCALAB 250Xi (Thermo Fisher Scientific, Waltham, MA, USA), which used an Al Kα source. The XPS data were calibrated for binding energy at C_1s_ = 248.8 eV. The morphology and element distribution of the slag were studied by a field emission scanning electron microscope (JSM-7800F, JEOL, Akishima, Japan), which was equipped with an Oxford SDD for EDS analysis (Oxford Instruments, High Wycombe, UK). The fluorine contents were determined by DETA titration (GB/T 18114.11-2010), with an analytical error of <0.4 wt.%. The total amount of rare earth oxides was determined by the oxalate precipitation method (GB/T 14635-2010), with an analytical error of <0.5 wt.%. The rare earth oxide relative contents were analyzed by ICP-OES (Agilent, Santa Clara, CA, USA) (GB/T 18114.8-2010), with an analytical error of <0.4 wt.%. The lithium content was detected by an inductively coupled plasma optical emission spectrometer (YS/T 739.3-2020), with an analytical error of <0.27 wt.%.

## 3. Results and Discussion

### 3.1. Analysis of Raw Material

**Figure 1:**
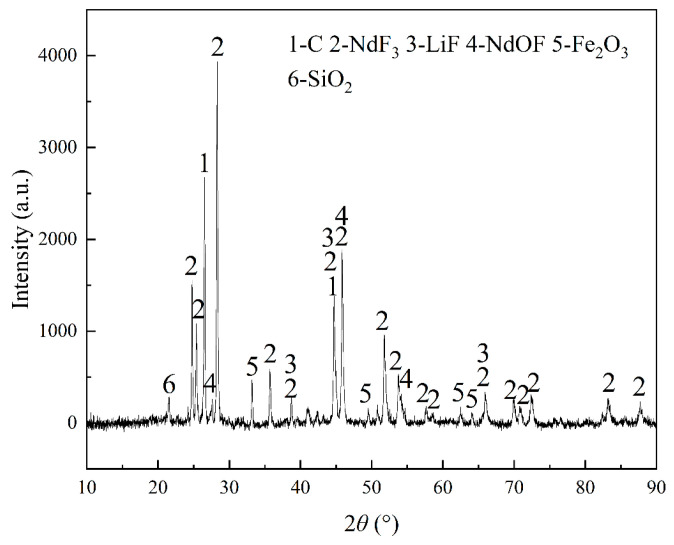
The XRD pattern of the raw material.

**Figure 2:**
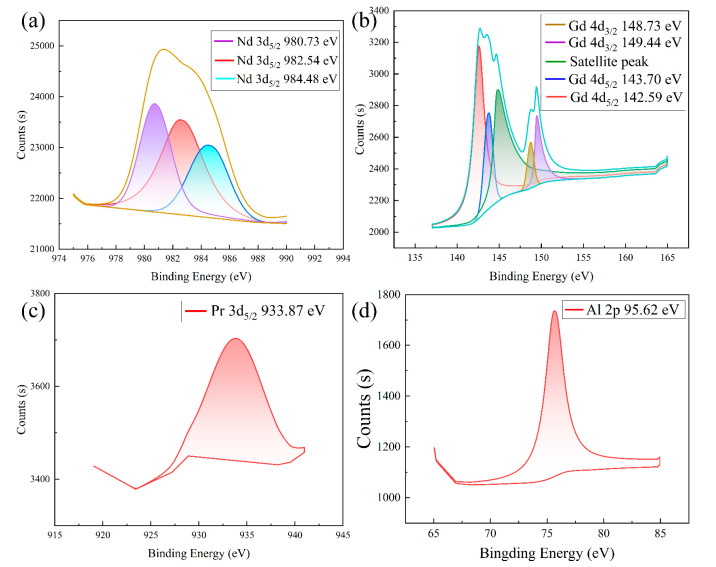
XPS results of the raw material: (**a**) Nd; (**b**) Gd; (**c**) Pr; (**d**) Al.

The composition of REMES is displayed in Table 1 and Table 2. In this slag, Nd, Pr, and Gd are the major REEs. Additionally, according to Figure 1, neodymium fluoride was the major phase of the REEs and neodymium oxyfluoride was also present, with some oxide impurities, including Fe_2_O_3_ and SiO_2_. As shown in Figure 2, the XPS analysis illustrated that neodymium compounds were mainly composed of NdF_3_ and that gadolinium was combined with both O and F, while praseodymium was combined with O. Notably, the XRD pattern shows no peak of Al, while the XPS result displays a peak of Al_2_O_3_, which might be caused by the detection limit of XRD.

**Figure 3:**
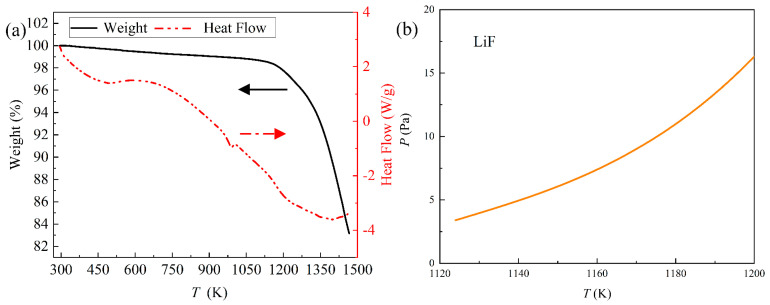
(**a**) The TG-DSC results of the raw material; (**b**) the saturated vapor pressure of LiF.

To determine the distillation temperature, the raw material was analyzed by TG-DSC. The results are shown in Figure 3a. The DSC curve showed an endothermic peak at approximately 950 K, and some substance might have melted at this temperature. Additionally, the weight change of REMES had no obvious difference until approximately 1100 K. Figure 3b shows the saturated vapor pressure of LiF calculated by the Antoine equation [33], which reaches 10 Pa before 1180 K, indicating that the evaporation of some substance might start at 1100 K. Therefore, the initial distillation temperature for experiments with different temperatures was selected as 1173 K.

In a previous study, the distillation temperature of NdF_3_ was estimated [34]. NdF_3_ could evaporate from the slag after 1473 K at 1 Pa. Compared with the saturated vapor pressure of LiF, NdF_3_ and LiF could condensate at different places.

In summary, the REMES was mainly composed of REEs, fluorine, carbon, lithium, and some oxide impurities. Rare earth elements primarily existed as fluorides, with some forms of oxyfluorides and oxides. Lithium was mainly lithium fluoride. The main components of the oxide impurities were silicon, iron, and aluminum oxides. Combined with the TG-DSC results and the thermodynamic analysis of NdF_3_ and LiF, the vacuum distillation method could be used to recycle this slag.

### 3.2. The Distillation Experiments of REMES and the Phase Changes of REEs

In this part, the REMES was directly distilled in the abovementioned self-made vertical furnace in a vacuum. The distillation results at different distillation times and temperatures were investigated. And the condensates were characterized to analyze the phase changes to find potential hurdles.

#### 3.2.1. The Distillation Experiments on the Distillation Time for the Phase Changes of REMES

**Figure 4:**
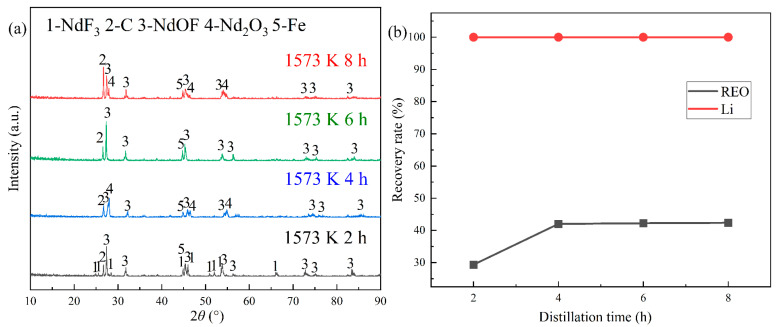
The consequences of the experiments at different distillation times: (**a**) the XRD patterns of the distillation residue; (**b**) the changes in the recovery efficiencies of the REEs and Li with time.

The REMES sample was heated at 1573 K and 0.1 Pa with the distillation time varying from 2 h to 8 h. The results are showcased in Figure 4. They suggest that when the distillation time was 2 h, the diffraction peaks of NdF_3_ significantly decreased, while those of NdOF still remained. After the distillation time reached 4 h, NdF_3_ disappeared and only NdOF could be observed. Additionally, the recovery of LiF could be completed at 2 h, and the rare earth recovery efficiency reached a maximum of 42.04% at 4 h (see Figure 4b). This phenomenon indicated that there were at least two kinds of phase transitions for the rare earth compounds during the distillation process, including the evaporation of rare earth fluorides and LiF as well as the generation of NdOF.

To completely investigate the transition of rare earth compounds, it was necessary to conduct experiments at different distillation temperatures. The distillation time was chosen as 6 h to ensure the complete transition.

#### 3.2.2. The Distillation Experiments on the Temperature for the Phase Changes of REMES

**Figure 5:**
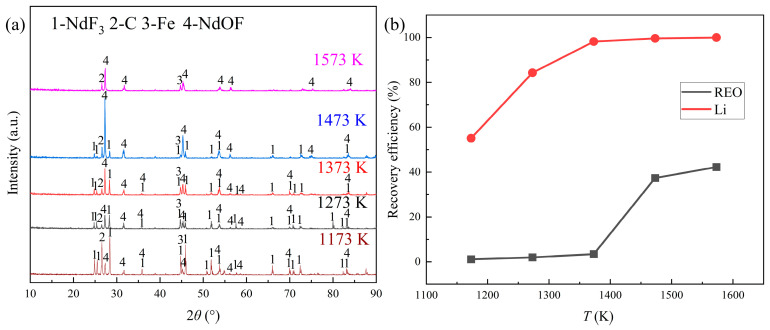
The results of the experiments at different distillation temperatures: (**a**) the XRD patterns of the distillation residue; (**b**) the changes in the recovery efficiencies of the REEs and Li with temperature.

The possible phase changes of rare earth fluorides were investigated through the distillation experiments of REMES at different temperatures. According to the TG-DSC results, the distillation temperature varied from 1173 K to 1573 K. The other conditions were a distillation time of 6 h and an absolute pressure of 0.1 Pa. The results are shown in Figure 5.

Figure 5a illustrates that the diffraction peaks of NdF_3_ gradually decreased, with the disappearance of NdF_3_ observed at 1573 K, while the rise in the diffraction peaks of NdOF started at 1173 K, reaching their highest points at 1473 K. According to Figure 5b, at 1173 K, only LiF could evaporate. When the temperature reached 1373 K, a high recovery efficiency of Li could be obtained, and the recovery efficiency of Li then increased slightly. As for the distillation of REEs, the evaporation of rare earth fluorides could not occur until 1473 K, with 37.36% of the total REEs recovered, and the rare earth recovery efficiency improved slightly at 1573 K.

The results were consistent with the results of the distillation time experiments. Additionally, the generation of NdOF might start before 1173 K, and this reaction notably affected the recovery of rare earth fluorides.

#### 3.2.3. Further Investigation on the Phase Transformation in the Distillation Process

**Figure 6:**
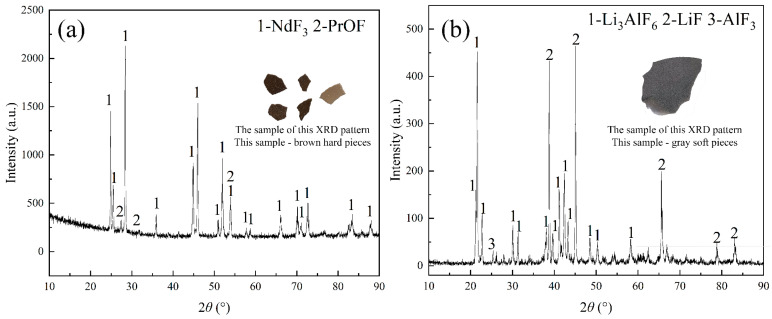
XRD patterns and corresponding sample pictures of the condensates at 1573 K for 6 h: (**a**) the rare earth fluoride condensate; (**b**) the lithium fluoride condensate.

To further investigate the phase transitions of REMES in the distillation process, the distillation condensates harvested at 1573 K for 6 h were analyzed. The results are shown in Figure 6.

In Figure 6a, NdF_3_ with a small amount of PrOF was found, indicating that the evaporation of rare earth fluorides occurred at 1573 K, and PrOF also possessed the ability to evaporate in a high-vacuum environment. Interestingly, AlF_3_ and lithium cryolite could be observed in the condensate containing LiF, which illustrated that it was difficult to obtain a high-purity LiF product. Notably, only Al_2_O_3_ was discovered in the raw material, suggesting that AlF_3_ and lithium cryolite were generated during the distillation process. These phenomena indicated that some oxide impurities might capture the fluorine from rare earth fluorides in the high-temperature and -vacuum environment, leading to the formation of REOF and RE_2_O_3_.

**Figure 7:**
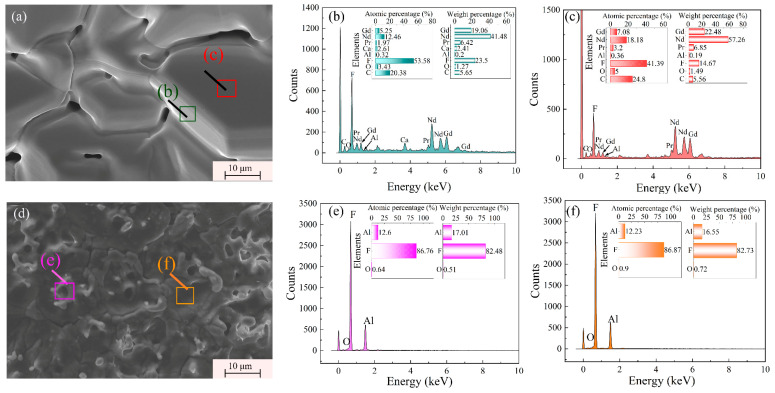
(**a**) SEM image of the rare earth condensate; (**b**,**c**) microdistribution of the rare earth condensate; (**d**) SEM image of the lithium condensate; (**e**,**f**) microdistribution of the Lithium condensate.

Figure 7 shows the morphology and microdistribution of the two condensates. Figure 7a shows the morphology of the condensate containing REEs, and the microdistributions of areas (b) and (c) exhibited that this condensate was mainly composed of rare earth fluorides with a small amount of Al and Ca. Figure 7e,f show the existence of aluminum and a high content of fluorine in the condensate containing Li. Although Li could not be observed by EDS, the high fluorine content suggested the possible presence of Li. These EDS results agreed with the corresponding XRD patterns. Therefore, there might be some reactions between REF_3_ and oxide impurities, resulting in the generation of REOF during the distillation process. The potential reactions are shown as Equations (2)–(12). Since the main rare earth element was Nd, the possible reactions of Nd were used as examples.

NdF_3_ + MgO = NdOF + MgF_2 _ (2)

NdF_3_ + CaO = NdOF + CaF_2_ (3)

3NdF_3_ + Fe_2_O_3_ = 3NdOF + 2FeF_3_(g) (4)

3NdF_3_ + Al_2_O_3_ = 3NdOF + 2AlF_3_(g) (5)

2NdF_3_ + SiO_2_ = 2NdOF + SiF_4_(g) (6)

NdF_3_ + Nd_2_O_3_ = 3NdOF (7)

2NdF_3_ + 3MgO = Nd_2_O_3_ + 3MgF_2_(g) (8)

2NdF_3_ + 3CaO = Nd_2_O_3_ + 3CaF_2_(g) (9)

2NdF_3_ + Fe_2_O_3_ = Nd_2_O_3_ + 2FeF_3_(g) (10)

2NdF_3_ + Al_2_O_3_ = Nd_2_O_3_ + 2AlF_3_(g) (11)

4NdF_3_ + 3SiO_2_ = 2Nd_2_O_3_ + 3SiF_4_(g) (12)

**Figure 8:**
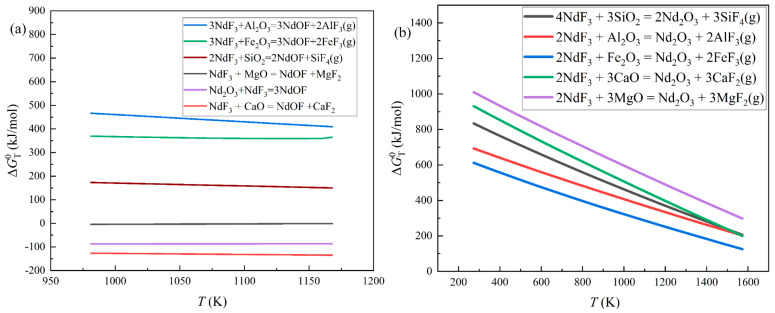
The standard Gibbs free energy changes of the reactions between oxide impurities and NdF_3_: (**a**) the changes in ${\Delta G}_{T}^{0}$ for Equations (2)–(7) with temperature; (**b**) the changes in ${\Delta G}_{T}^{0}$ for Equations (8)–(12) with temperature.

Equations (2)–(12) are the possible reactions in the distillation period. Due to the data of NdOF, Equations (2)–(7) were calculated according to the data of Turdogan and Ji, while Equations (8)–(12) were computed by HSC 6.0 software [35,36]. The results are shown in Figure 8.

Figure 8a exhibits the standard Gibbs free energy changes of Equations (2)–(7). Al_2_O_3_, Fe_2_O_3_, and SiO_2_ are difficult to react with NdF_3_, while NdOF might be generated by the reactions of MgO and CaO. Additionally, NdOF could also be produced by the reactions between NdF_3_ and Nd_2_O_3_. Figure 8b shows that the standard Gibbs free energy changes of Equations (8)–(12) will decrease with increasing temperature, while the changes are higher than 0 kJ/(mol) at 1573 K, which indicates that the reactions might have difficulty occurring at standard conditions.

**Figure 9:**
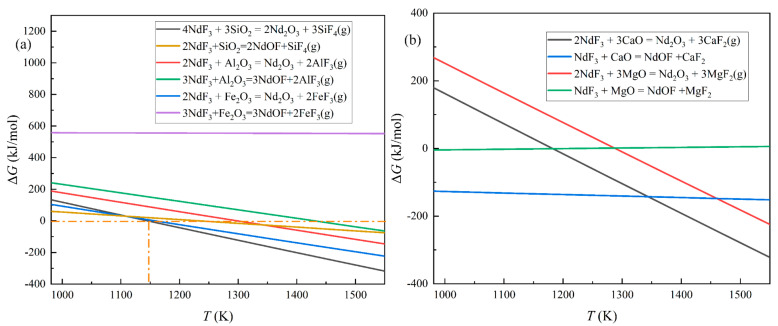
The Gibbs free energy changes of the reactions between the oxide impurities and NdF_3_ at 0.1 Pa: (**a**) the changes in ΔG for reactions of SiO_2_, Al_2_O_3_, and Fe_2_O_3_ with temperature; (**b**) the changes in ΔG for reactions of CaO and MgO with temperature.

Considering that the experiments were implemented in the nonstandard state, the Gibbs free energy changes were computed by thermodynamic isothermal equations, which are showcased in Equations (13) and (14). The calculation results are shown in Figure 9.

(13) $$\Delta G={\Delta G}_{T}^{0}+RTlnJ$$

(14) $$J={\left(\frac{P}{P^{0}}\right)}^{n}$$

where ${\Delta G}_{T}^{0}$ is the standard Gibbs free energy change of the reaction, kJ/(mol); *T* is the reaction temperature, K; *P* is the partial pressure of the gaseous product in the reaction, Pa; *n* is the stoichiometric number of the gas resultant; R is the gas constant, kJ/(mol·K); and P^0^ is the standard pressure, Pa.

Because the pressure of the experiments was 0.1 Pa, the partial pressure was also set to 0.1 Pa. In the nonstandard state, NdOF could be generated preferentially by reactions involving CaO and MgO until approximately 1350 K, after which Nd_2_O_3_ would appear in the reactions between oxide impurities and NdF_3_. However, according to Equation (7), the combination of NdF_3_ and Nd_2_O_3_ would occur, leading to the reappearance of NdOF again, which agrees with the enhancement of the NdOF diffraction peaks in Figure 5.

The calculation results and the experimental outcomes elucidated that oxide impurities could capture the fluorine from rare earth fluorides, transforming the rare earth fluorides into rare earth oxides and rare earth oxyfluorides. This process disturbed the evaporation of rare earth fluorides and resulted in a low recovery efficiency of REEs. Above all, the primary obstacle in vacuum distillation was oxide impurities. Therefore, eliminating oxide impurities was a significant mission for improving the efficiency of the distillation recovery method.

### 3.3. The Effect of the Fluorination Process on the Recovery of REMES by Vacuum Distillation

In a previous study, NH_4_HF_2_ was expected to be a potential fluorination reagent for rare earth oxides and oxyfluorides [34]. The additional fluorine sources of NH_4_HF_2_ might have the ability to transfer oxide impurities to the fluorides. Therefore, the fluorination process and the vacuum distillation were investigated.

#### 3.3.1. The Volatility of Fluoride Impurities

**Figure 10:**
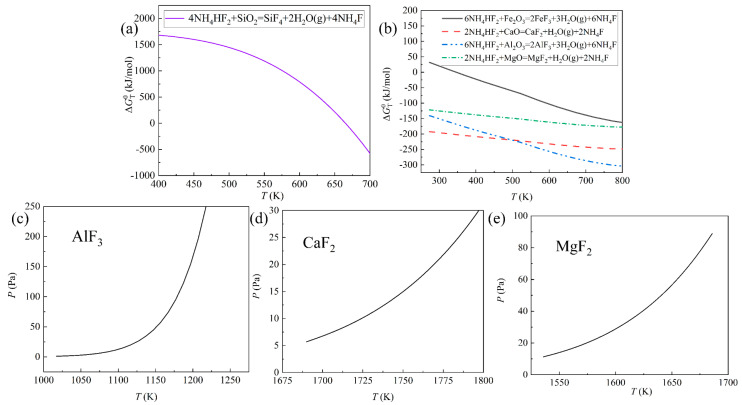
(**a**) The standard Gibbs free energy change of the reaction between SiO_2_ and NH_4_HF_2_; (**b**) the standard Gibbs free energy change of the reactions between oxide impurities and NH_4_HF_2_; (**c**–**e**) the saturated vapor pressure of AlF_3_, CaF_2_, and MgF_2_.

According to Figure 10a,b, NH_4_HF_2_ also possesses the ability to fluorinate oxide impurities. Therefore, in order to detect the distillation temperature of fluoride impurities, the volatility of them should be studied.

The reactions in Figure 10a,b were calculated by HSC 6.0 software. Additionally, the saturated vapor pressures of AlF_3_, CaF_2_, and MgF_2_ were obtained from a book called “*The Yaws Handbook of Yapor Pressure Antoine Coefficients*” and calculated by the Antoine equation [32]. Due to the lack of parameters, the interference of FeF_3_ in the distillation of rare earth fluoride was examined in the distillation experiment.

SiF_4_ is a gaseous substance, so it is separated during the fluorination process. As shown in Figure 6 and Figure 10c, AlF_3_ easily evaporates and combines with LiF. The saturated vapor pressures of CaF_2_ and MgF_2_ indicate that the distillation temperature of CaF_2_ and MgF_2_ might be higher than or around 1573 K, which might affect the purity of rare earth fluorides (see Figure 10d,e). Due to the low content of CaO and MgO and the evaporation of rare earth fluorides at 1473 K, the separation of CaF_2_ and MgF_2_ could be disregarded. Therefore, fluorinating REMES was deemed feasible.

#### 3.3.2. Treatment for REMES by Fluorination and Vacuum Distillation

To fluorinate the oxide impurities and rare earth compounds, 20 g of NH_4_HF_2_ was utilized to react with 10 g of REMES. The materials were mixed in a graphite crucible and placed in an airtight vertical furnace with a cover. To fulfill the fluorination reaction, the fluorination experiment was conducted at 773 K for 6 h.

**Figure 11:**
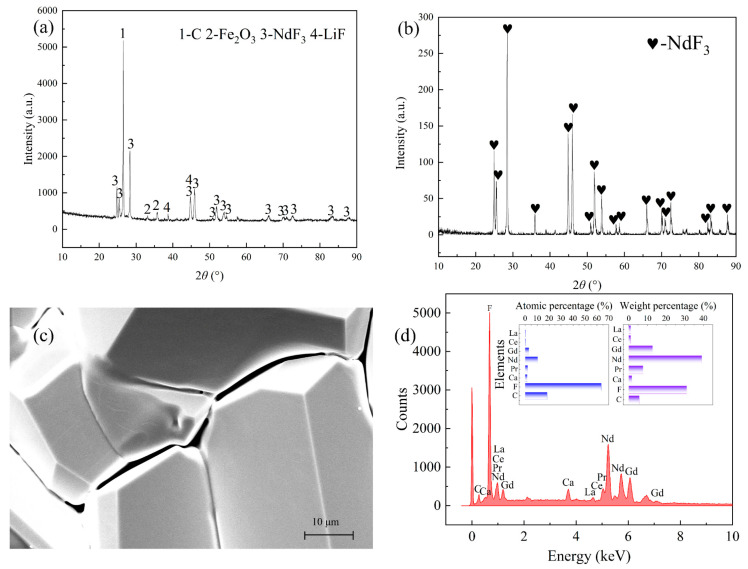
The fluorination and distillation results: (**a**) the fluorinated slag; (**b**) the distillation condensate of the REEs; (**c**) SEM image of the rare earth condensate; (**d**) EDS data of the rare earth condensate.

As shown in Figure 11, the diffraction peaks of NdOF and Nd_2_O_3_ disappeared in the XRD pattern of the fluorinated REMES. In addition, the fluorine content increased to 30.64 wt.%. Then, the fluorinated slag was compacted and then distilled at 1573 K and 0.1 Pa for 6 h. The recovery efficiencies of the REEs and Li were calculated by Equation (1), and the results are displayed in Table 3.

According to the experimental results, the recovery efficiency of the REEs increased to 86.23%. Additionally, the XRD pattern also confirmed the high purity of the rare earth product. Although CaF_2_ could still be found in the product by SEM-EDS, the purity of the rare earth product was adequate. Notably, Fe_2_O_3_ could still be observed in the fluorinated slag, indicating that it was difficult to fluorinate Fe_2_O_3_ in the slag and that the existing Fe_2_O_3_ might be the main obstacle for the distillation of rare earth fluoride. Furthermore, as shown in Figure 5a, the peaks of Fe in the XRD pattern of the distillation residue indicated that Fe_2_O_3_ would be reduced by carbon during the distillation process, which might decrease the effect of Fe_2_O_3_ on the distillation of rare earth fluorides. Therefore, NH_4_HF_2_ could indeed increase the recovery efficiency of REEs and reduce the influence of impurities on the product purity.

Additionally, a condenser was installed at the top of the furnace, and most of the gaseous fluorides could condense on this condenser, including SiF_4_. Figure S1 shows that SiF_4_ could be captured by NH_4_F and condensed as (NH_4_)_2_SiF_6_ instead of being released into the air. Therefore, the potential pollution can be addressed.

Overall, NH_4_HF_2_ could transfer the oxide impurities as well as rare earth oxides and oxyfluorides with most gaseous fluorides recovered in the condenser, but only a small amount of CaF_2_ could affect the purity of the rare earth condensate.

#### 3.3.3. Comparison with Other Literature

This fluorination–vacuum distillation methodology is a new method in the REMES recovery field. Compared with other methodologies, this method possesses its own advantages, while some weaknesses still exist. The comparison is shown in Table 4.

Compared with other research, the clean and short process is a unique advantage of this method, while high energy consumption and high cost of NH_4_HF_2_ must be considered. Because the high temperature is essential for vacuum distillation, a search for low-cost fluorination reagents is an important mission to implement besides the investigation of optimal conditions.

## 4. Conclusions

This study introduced the phase transition of REMES in high-temperature and -vacuum conditions, elucidating the obstacles to recovering REEs by vacuum distillation. And then, the investigation for the fluorination process was implemented. The main conclusions were as follows:

(1) The distillation experimental results showed that 42.04% of the rare earth fluorides and 99.99% of the lithium fluoride in the rare earth molten salt electrolytic slag could be evaporated at 1573 K and 0.1 Pa for 4 h, and the rare earth oxides as well as the rare earth oxyfluorides would be generated simultaneously.
(2) The phase transition analysis elucidated that the oxide impurities could react with rare earth fluorides under high-temperature and -vacuum conditions, capturing the fluorine element and generating rare earth oxides as well as oxyfluorides. This phenomenon significantly affected the recovery efficiency of REEs.
(3) The fluorination experiments indicated that the fluorination process could fluorinate both rare earth compounds and oxide impurities, and after fluorinating the slag by 20 g NH_4_HF_2_ at 773 K for 6 h, the recovery efficiency of the rare earth elements could increase to 86.23% at 1573 K and 0.1 Pa for 6 h, while some problems, such as the fluorination of Fe_2_O_3_, still existed.

In this paper, the phase change analysis discovered the obstacle to applying vacuum distillation, and fluorination was selected as the solution for the obstacle, proving the feasibility of REE recovery by vacuum distillation. The vacuum distillation method is an environmentally friendly method, possessing the potential of recovering resources with no wastewater or gas production. Although further investigation is still necessary, this study also hopes that this attempt at vacuum distillation could inspire readers, making it possible to put an environmentally friendly treatment into practice.

## Figures and Tables

**Table 1:** The composition of rare earth molten salt electrolytic slag.

Composition	REEs (Calculated as Oxides)	F	Li	TFe	Al_2_O_3_	SiO_2_	CaO	MgO	C
Weight percent/%	50.67	19.52	3.05	4.67	3.31	5.15	0.63	0.58	15.10

**Table 2:** The composition of REEs in the REMES.

Composition (Calculated as Oxides)	Nd	Pr	Gd	Ce	La	Dy
Weight percent/%	69.07	14.19	14.33	0.31	0.89	1.21

**Table 3:** The recovery efficiencies of REEs and Li.

Elements	REEs (%)	Li (%)
Recovery efficiency	86.23	99.99

**Table 4:** The comparison between the fluorination–vacuum distillation method and other techniques.

Method	Procedures	Advantage	Disadvantages	Reference
Fluorination–Vacuum Distillation Method	(1) Fluorination roasting; (2) Vacuum distillation.	(1) Short process; (2) Clean process; (3) High resource utilization.	(1) High energy consumption; (2) High cost of NH4HF2.	
Fluoride Sulfate Conversion Method	(1) Magnetic separation; (2) Roasting with sulfuric acid; (3) Absorbing HF; (4) Fluorination precipitation.	(1) Low energy consumption; (2) Low production cost; (3) High resource utilization.	(1) Wastewater problem; (2) The corrosion effect of HF.	Tian et al. [27]
Roasting Activation Method	(1) Activation roasting; (2) Sulfuric acid leaching; (3) Hierarchical extraction.	(1) Low energy consumption; (2) Low production cost; (3) High resource utilization;	(1) Long process of solvent extraction procedure; (2) Wastewater problem.	Tong et al. [19]
Sub-molten salt Method	(1) NaOH sub-molten salt decomposition; (2) Hydrochloric acid leaching; (3) Oxalate precipitation; (4) Roasting to recover RE2O3; (5) Heating to recover Li and F and recycle NaOH and Na_2_CO_3_.	(1) Low energy consumption; (2) Low production cost.	(1) Long process; (2) Wastewater problem; (3) Impurity enrichment.	Yang et al. [25]
Borax Roasting–Hydrochloric Acid Leaching Method	(1) Roasting with borax; (2) Washing in the NaOH solution; (3) Hydrochloric acid leaching; (4) Recycling of NaOH and F; (5) Solvent extraction to recover REEs.	(1) Low energy consumption; (2) Low production cost.	(1) Long process of solvent extraction procedure; (2) Wastewater problem; (3) Low resource recovery efficiency of lithium.	Yang et al. [26]

## Data Availability

The original contributions presented in the study are included in the article. Further inquiries can be directed to the corresponding authors.

## Supplementary Materials

The following supporting information can be downloaded at: https://www.mdpi.com/article/10.3390/ma18071538/s1, Figure S1: The condensate in the fluorination process.

## References

[1] Gao Z., Xu Y., Qi Y., Feng Z., Dong B. (2025). Microenvironment regulation of the electronic structure of bismuth oxychloride via rare-earth element samarium doping for remarkable visible-light-responsive oxygen evolution. Appl. Surf. Sci..

[2] Lee S., Kim G., Lee K., Kim S., Kim T., Lee S., Kim D., Lee W., Lee J. (2025). A novel two-step grain boundary diffusion process using TaF_5_ and Pr_70_Cu_15_Al_10_Ga_5_ for realizing high-coercivity in Nd-Fe-B-sintered magnets without use of heavy rare-earth. Acta Mater..

[3] Asadullayeva S.G., Ismayilova N.A. (2024). Effect of rare earth ions on the optical and electronic properties of defect chalcopyrite: Experimental and theoretical investigation. Solid. State Commun..

[4] Navarro-Espinoza S., Meza-Figueroa D., Meléndrez-Amavizca R., Barboza-Flores M., Soto-Puebla D., Ruiz-Torres R., Silva-Campa E., Paz-Moreno F. (2025). The role of rare earth elements in three-way catalysts: Implications for automobile emission control. Ceram. Int..

[5] Patil A., Thalmann N., Torrent L., Tarik M., Struis R., Ludwig C. (2023). Surfactant-based enrichment of rare earth elements from NdFeB magnet e-waste: Optimisation of cloud formation and rare earths extraction. J. Mol. Liq..

[6] Ghorbani Y., Ilankoon I., Dushyantha N., Nwaila G. (2025). Rare earth permanent magnets for the green energy transition: Bottlenecks, current developments and cleaner production solutions. Resour. Conserv. Recycl..

[7] Gad S., Jin Z., Emad S., Vergara J., Yawas D., Dagwa I., Omiogbemi I. (2023). Potential of rare-earth compounds as anticorrosion pigment for protection of aerospace AA2198-T851 alloy. Heliyon.

[8] US Geological Survey (2024). P Mineral Commodity Summaries 2024.

[9] US Geological Survey (2020). P Mineral Commodity Summaries 2020.

[10] Omodara L., Pitkaaho S., Turpeinen E., Saavalainen P., Oravisjarvi K., Keiski R. (2019). Recycling and substitution of light rare earth elements, cerium, lanthanum, neodymium, and praseodymium from end-of-life applications—A review. J. Clean. Prod..

[11] Liang B., Gu J., Zeng X., Yuan W., Rao M., Xiao B., Hu H. (2024). A Review of the Occurrence and Recovery of Rare Earth Elements from Electronic Waste. Molecules.

[12] Xiao F., Hu W., Zhao J., Zhu H. (2023). Technologies of Recycling REEs and Iron from NdFeB Scrap. Metals.

[13] Wang L., Chen Y., Tso Y., Sheng C., Ponou J., Kou M., Zhou H., Chen W. (2020). Separation of Cerium Oxide Abrasive and Glass Powder in an Abrasive-Glass Polishing Waste by Means of Liquid–Liquid–Powder Extraction Method for Recovery: A Comparison of Using a Cationic and an Anionic Surfactant Collector. Sustainability.

[14] Borra C.R., Vlugt T.H., Yang Y., Offerman S.E. (2018). Recovery of Cerium from Glass Polishing Waste: A Critical Review. Metals.

[15] Daminescu D., Duteanu N., Ciopec M., Negrea A., Negrea P., Nemeş N.S., Pascu B., Lazău R., Berbecea A. (2023). Kinetic Modelling the Solid–Liquid Extraction Process of Scandium from Red Mud: Influence of Acid Composition, Contact Time and Temperature. Materials.

[16] Panda S., Costa R., Shah S., Mishra S., Bevilaqua D., Akcil A. (2021). Biotechnological trends and market impact on the recovery of rare earth elements from bauxite residue (red mud)—A review. Resour. Conserv. Recycl..

[17] Pérez-Cardona J., Huang T., Zhao F., Sutherland J., Atifi A., Fox R., Baek D. (2022). Molten salt electrolysis and room temperature ionic liquid electrochemical processes for refining rare earth metals: Environmental and economic performance comparison. Sustain. Energy Technol. Assess..

[18] Chen L., Li Z., Gong A., Tian L., Xu Z. (2019). Research Progress of Rare Earth Recovery from Rare Earth Waste. J. Chin. Rare Earth Soc..

[19] Tong Z., Hu X., Wen H. (2023). Effect of roasting activation of rare earth molten salt slag on extraction of rare earth, lithium and fluorine. J. Rare Earth.

[20] Liang Y., Li Y., Xue L., Zou Y. (2018). Extraction of rare earth elements from fluoride molten salt electrolytic slag by mineral phase reconstruction. J. Clean. Prod..

[21] Mubula Y., Yu M., Yang D., Niu H., Qiu T., Mei G. (2024). Recovery of Rare Earths from Rare-Earth Melt Electrolysis Slag by Mineral Phase Reconstruction. JOM.

[22] Xing P., Li H., Ye C., Zhong L. (2022). Recovery of Rare-Earth Elements from Molten Salt Electrolytic Slag by Fluorine Fixation Roasting and Leaching. J. Sustain. Metall..

[23] Hu H., Wang J. (2021). Selective extraction of rare earths and lithium from rare earth fluoride molten-salt electrolytic slag by nitration. Hydrometallurgy.

[24] Wu H., Yan H., Liang Y., Qiu S., Zhou X., Zhu D., Qiu T. (2023). Rare earth recovery from fluoride molten-salt electrolytic slag by sodium carbonate roasting-hydrochloric acid leaching. J. Rare Earth.

[25] Yang D., Yu M., Mubula Y., Yuan W., Huang Z., Lin B., Mei G., Qiu T. (2024). Recovering rare earths, lithium and fluorine from rare earth molten salt electrolytic slag using sub-molten salt method. J. Rare Earth.

[26] Yang Y., Wei T., Xiao M., Niu F., Shen L. (2019). Rare Earth Recovery from Fluoride Molten-Salt Electrolytic Slag by Borax Roasting-Hydrochloric Acid Leaching. JOM.

[27] Tian L., Chen L., Gong A., Wu X., Cao C., Xu Z. (2021). Recovery of rare earths, lithium and fluorine from rare earth molten salt electrolytic slag via fluoride sulfate conversion and mineral phase reconstruction. Miner. Eng..

[28] Mubula Y., Yu M., Gu H., Wang L., Chen M., Qiu T., Mei G. (2024). Recovery of rare earths from rare earth molten salt electrolytic slag using alkali phase reconstruction method with the aid of external electric field. J. Rare Earth.

[29] Mubula Y., Yu M., Yang D., Niu H., Gu H., Qiu T., Mei G. (2024). Microwave-assisted atmospheric alkaline leaching process and leaching kinetics of rare earth melt electrolysis slag. Heliyon.

[30] Zhang M., He B., Liu Y., Xu L., Liang Y. (2024). Recovery of rare earth elements from rare earth molten salt electrolytic slag via fluorine fixation by MgCl_2_ roasting. J. Rare Earth.

[31] Lai Y., Li J., Zhu S., Liu K., Xia Q., Huang M., Hu G., Zhang H., Qi T. (2023). Recovery of rare earths, lithium, and fluorine from rare earth molten salt electrolytic slag by mineral phase reconstruction combined with vacuum distillation. Sep. Purif..

[32] Xu G. (1995). Rare Earths.

[33] Yaws C. (2015). The Yaws Handbook of Yapor Pressure Antoine Coefficients.

[34] Yang Z., Xiao F., Sun S., Zhong H., Tu G. (2024). REEs recovery from molten salt electrolytic slag: Challenges and opportunities for environmentally friendly techniques. J. Rare Earth.

[35] Ji C., Xi Z. (1990). Standard Gibbs Free Energy of Formation of Neodymium Oxyfluoride and Borates. J. Less-Common. Met..

[36] Turdogan E.T. (1988). Physical Chemistry of High Temperature Technology.

