# Supplementary figures and images for: Investigation on the Recovery of Rare Earth Fluorides from Spent Rare Earth Molten Electrolytic Slag by Vacuum Distillation
Source: Materials (Basel). 2025 Mar 28;18(7):1538. doi: 10.3390/ma18071538 (PMC11990493; doi:10.3390/ma18071538)

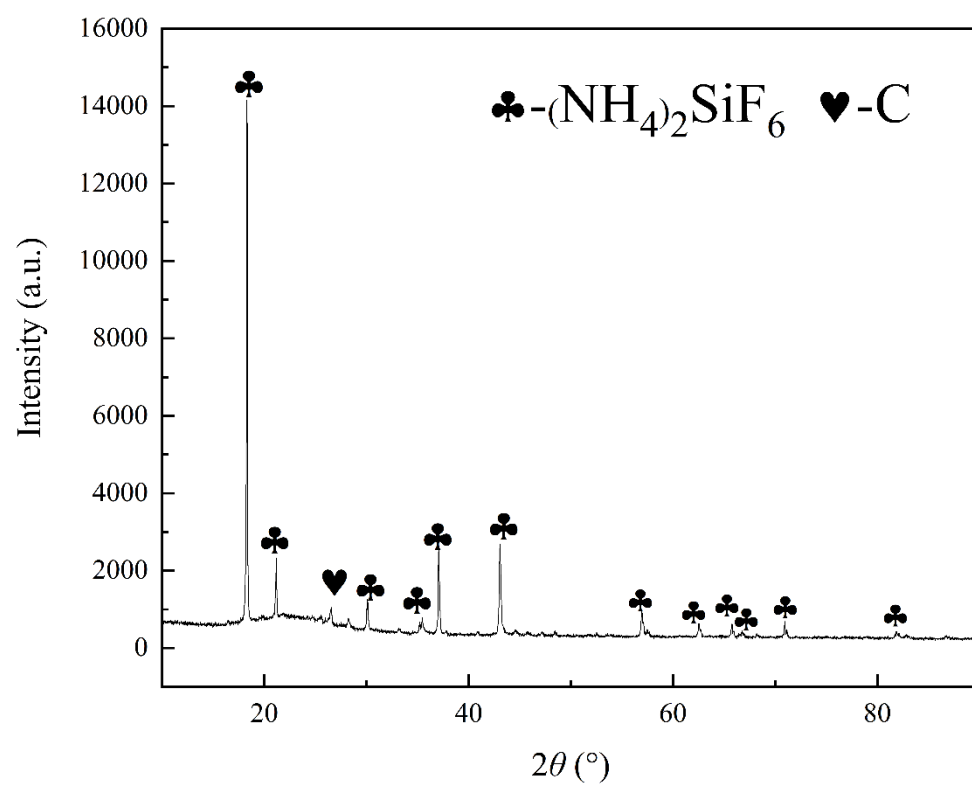

**Figure S1.** The condensate in the fluorination process

Supplement: Supplementary file 1 [file materials-18-01538-s001.zip › materials-3506619-supplementary.pdf]
